# Current knowledge and future directions of TLR and NOD signaling in sepsis

**DOI:** 10.1186/s40779-014-0029-7

**Published:** 2015-01-07

**Authors:** Niamh M Foley, Jian Wang, H Paul Redmond, Jiang Huai Wang

**Affiliations:** Department of Academic Surgery, University College Cork, Cork University Hospital, Cork, Ireland; Department of Pediatric Surgery, Affiliated Children’s Hospital, Soochow University, Suzhou, 215003 China

**Keywords:** Innate immunity, TLR signaling, NOD signaling, Sepsis

## Abstract

The incidence of sepsis is increasing over time, along with an increased risk of dying from the condition. Sepsis care costs billions annually in the United States. Death from sepsis is understood to be a complex process, driven by a lack of normal immune homeostatic functions and excessive production of proinflammatory cytokines, which leads to multi-organ failure. The Toll-like receptor (TLR) family, one of whose members was initially discovered in *Drosophila,* performs an important role in the recognition of microbial pathogens. These pattern recognition receptors (PRRs), upon sensing invading microorganisms, activate intracellular signal transduction pathways. NOD signaling is also involved in the recognition of bacteria and acts synergistically with the TLR family in initiating an efficient immune response for the eradication of invading microbial pathogens. TLRs and NOD1/NOD2 respond to different pathogen-associated molecular patterns (PAMPs). Modulation of both TLR and NOD signaling is an area of research that has prompted much excitement and debate as a therapeutic strategy in the management of sepsis. Molecules targeting TLR and NOD signaling pathways exist but regrettably thus far none have proven efficacy from clinical trials.

## Introduction

Sepsis remains a significant global burden, notwithstanding the noteworthy breakthroughs that have been made in our understanding of the molecular mechanisms involved. Sepsis has been identified as one of five conditions accounting for the most costly hospital length of stays in the United States [1]. The body’s response to sepsis is both intricate and diverse. Since the initial finding of the Toll-like receptor (TLR) in *Drosophila*, mapping of the pathways involved in the immune response to sepsis has grown significantly. TLRs and NOD-like receptors (NLRs) are the two main members of the family of pattern recognition receptors (PRRs) and are involved in the recognition of invading microbial pathogens. Both TLR and NOD signaling act synergistically in the initiation of the host innate immune response to bacterial infection. This involves the activation of intracellular signal transduction pathways and culminates in the production and release of proinflammatory cytokines. The targeting of these molecules and pathways presents potential therapeutic strategies in the treatment of sepsis. Unfortunately, to date, there have been no commercially available successful strategies in targeting the molecular pathways involved in sepsis. Disparities in the patient population and comorbid conditions complicate the development of successful strategies targeting various steps in the signaling pathways. In this review article we aim to highlight research to date on TLR and NOD signaling in sepsis and therapeutic strategies targeting these pathways.

### Incidence and mortality of sepsis

Sepsis is defined as the presence (probable or documented) of infection together with systemic manifestations of infection [2]. Several different terms have been used to describe the overwhelming inflammatory response associated with acute infections including septicemia, sepsis and septic shock. Considering the wide range of terms and the absence of tangible definitions, a consensus meeting was convened in 1992. The American College of Chest Physicians/Society for Critical Care Medicine (ACCP/SCCM) [3] sepsis definitions from this conference described sepsis as the clinical or microbiological evidence of infection along with two out of four of the following parameters:

- Temperature >38°C or <36°C;
- Heart rate >90 bpm;
- Hyperventilation >20 resps/min or Pa_CO2_ < 32 mmHg;
- White cell counts >12,000 cells/μl or <4,000 cell/μl.

These criteria were subsequently updated in the Society of Critical Care Medicine/European Society of Intensive Care Medicine/American College of Chest Physicians/American Thoracic Society/Surgical Infection Society (SCCM/ESICM/ACCP/ATS/SIS) International Sepsis Definitions Conference in 2001 (Table 1) [4]. The range of criteria included reflects the difficulty that has existed in the definition of sepsis. Sepsis is a systemic, deleterious host response to infection leading to severe sepsis and septic shock [5].

**Table 1:** **Diagnostic criteria for sepsis reproduced from the Society of Critical Care Medicine/European Society of Intensive Care Medicine/American College of Chest Physicians/American Thoracic Society/Surgical Infection Society (SCCM/ESICM/ACCP/ATS/SIS) International Sepsis Definitions Conference in 2001**

**Infection** ^**(1)**^ **: Documented or suspected and some of the following** ^**(2)**^	
**General parameters**	Fever (core temperature >38.3°C)
	Hypothermia (core temperature <36°C)
	Heart rate >90 beats per minute or >2 SD above the normal value for age
	Tachypnoea >30 breaths per minute
	Altered mental state
	Significant oedema or positive fluid balance (>20 ml/kg over 24 hrs)
	Hyperglycaemia (plasma glucose >110 mg/dL or 7.7 mmol/L in the absence of diabetes
**Inflammatory parameters**	Leukocytosis (white blood cell count >12,000/μL)
	Leukopaenia (white blood cell count <4,000/μL)
	Normal white blood cell count with >10% immature forms
	Plasma C reactive protein >2 SD above the normal value
	Plasma procalcitonin >2 SD above the normal value
**Haemodynamic parameters**	Arterial hypotension^(2)^ (systolic blood pressure <90 mmHg, mean arterial pressure <70 mmHg or a systolic blood pressure decrease >40 mmHg in adults or <2 SD below the normal value for age)
	Mixed venous oxygen saturation >70%^(2)^
	Cardiac index >3.5 L/min/m^2(3,4)^
**Organ dysfunction parameters**	Arterial hypoxaemia (PaO_2_/FiO_2_ <300)
	Acute oliguria (urine output <0.5 ml/kg/hr or 45 mmol/L for at least 2 hrs)
	Creatinine increase ≥0.5 mg/dL
	Coagulation abnormalities (international normalised ratio >1.5 or activated partial thromboplastin time >60 seconds)
	Ileus (absent bowel sounds)
	Thrombocytopenia (platelet count <100,000/μL)
	Hyperbilirubinaemia (plasma total bilirubin >4 mg/dL or 70 mmol/L)
**Tissue perfusion parameters**	Hyperlactatemia (>3 mmol/L)
	Decreased capillary refill or mottling

^(1)^Defined as a pathological process induced by microorganisms.

^(2)^Values above 70% are normal in children and should therefore not be used as a sign of sepsis in newborns or children.

^(3)^Values of 3.5-5.5 are normal in children and should therefore not be used as a sign of sepsis in newborns or children.

^(4)^Diagnostic criteria for sepsis in the paediatric population is signs and symptoms of inflammation plus infection with hyper- or hypothermia rectal temperature >38.5°C or <35°C, tachycardia (may be absent in hypothermic patients) and at least one of the following indications of altered organ function, altered mental status, hypoxemia, elevated serum lactate levels, and bounding pulses.

In the United States severe sepsis is the tenth leading overall cause of death, similar to the numbers dying from acute myocardial infarction. The risk of dying from sepsis is rising year on year [6,7]. Mortality associated with severe sepsis is estimated at 30%-50% [8,9]. Treatment in the initial hours after the onset of sepsis influences outcome. Significant variability exists in reported severe sepsis mortality with a rate of 8.6% (range 0.9%-18.2%) across 188 hospitals in the United States [10], though a further study reported higher in-hospital mortality rates ranging from 14.7%-29.9% [11]. Sepsis is a global financial burden [1]. The costs associated with sepsis care are mainly related to the price of targeted new therapies such as activated protein C, which costs $27,936 per life year gained [12], technologies and also the increasing charges for fixed costs. Angus and coworkers estimated the cost of sepsis treatment in the United States in 2001 at $16.7 billion annually [13]. This figure had risen to $24.3 billion by 2007 [8].

The vast majority of severe sepsis cases are as a result of infection with either gram-positive or gram-negative bacteria. The incidence of gram-positive sepsis has increased over time and is now almost as common as gram-negative sepsis [14]. In a 2006 European multicentre study, a respiratory source (68%) of sepsis was the commonest site involved, followed by an intra-abdominal source (22%) [15]. The same study revealed the most common isolate as *Staphylococcus aureus*, followed by *Pseudomonas species* and *Escherichia coli.* The Extended Study on the Prevalence of Infection in Intensive Care (EPIC II) also found a predominant respiratory source of sepsis (64%), with 62% of isolated bacteria being gram-negative microorganisms [16]. The most commonly isolated gram-negative bacteria were *Pseudomonas species*, *Escherichia coli* and *Klebsiella species. Staphylococcus aureus* was one of the most commonly isolated gram-positive bacteria followed by *Staphylococcus epidermidis* and *Streptococcus pneumonia*. The same study, using multivariate analysis, found that gram-negative bacteria, namely *Pseudomonas*, *Enterococcus* and *Acinetobacter* species, were associated with a greater risk of in-hospital mortality. The increasing incidence of gram-positive bacterial infection is possibly a result of the increasing numbers of invasive procedures and the increasing risk of developing hospital acquired infections [17].

Sir William Osler noted that death from sepsis results from the response of the body to systemic infection as opposed to the infection itself. This view was expanded on in the 1970’s and is now a widely accepted concept [18]. Death in the first few days from sepsis is generally understood to be a result of hyper-inflammation driven by inflammatory cytokines, which leads to multi-organ failure. Herein lies a complex dysregulation of the immune system with loss of immune homeostasis.

### Host innate immunity and related innate immune responses to microbial infection

The immune system affords the host an opportunity to respond to pathogenic microorganisms and incorporates innate and adaptive immunity. Innate immunity is a generic response to the recognition of invading microbial pathogens and defined as being “dependent on germline genes, present at all times and functional during early primary infections but not increasing with repeated exposure [19]”. By contrast, adaptive immunity is dependent on the “rearrangement of genes, antigen specific and requiring time for induction during primary challenges [19]”. Therefore, the innate immune system forms the first line of defense against microbial infection, and is activated by the engagement of germline-encoded innate immune receptors, also known as pattern recognition receptors (PRRs) in response to invading microbial pathogens [5,20,21]. PRRs expressed on innate immune cells such as polymorphonuclear neutrophils (PMNs) and monocytes/macrophages recognize the presence of highly conserved and unique structures of microbial pathogens called pathogen-associated molecular patterns (PAMPs) as well as detect endogenous damage-associated molecular patterns (DAMPs) generated in the setting of cellular damage or tissue injury [20,22,23]. This generic response enables the detection of a finite number of molecules that are common and conserved in different pathogenic microbes, for example lipopolysaccharide (LPS) or lipid A that is common to all gram-negative bacteria and lipoteichoic acid (LTA) that is common to all gram-positive bacteria. Recognition of these PAMPs by PRRs during microbial infection results in activation of signal transduction pathways and initiates both inflammatory and antimicrobial responses, which ultimately culminate in eliminating the invading microbial pathogens [5,20,21,24].

Monocytes/macrophages, PMNs and dendritic cells (DCs) are all salient elements of the host innate immune response. Natural killer cells, once thought to be solely a component of the innate immune system are now recognized to possess features associated with adaptive immunity also. These cells can directly or indirectly target pathogenic microorganisms through phagocytosis or by releasing substances such as inflammatory cytokines, chemokines and other mediators. Macrophages, derived from peripheral blood mononuclear cells, are predominantly resident in tissues and are particularly abundant in liver, lung and gut tissue, where the body is exposed to most foreign pathogens. Phagocytosis of invading microbial pathogens by macrophages initiates the innate immune response. The inflammatory response to invading pathogens is characterized by the release of a variety of different inflammatory mediators, including proinflammatory cytokines and chemokines, adhesion molecules, reactive oxygen species (ROS) and enzymes. This vital step in the elimination of microbial pathogens from the host can result in an overwhelming inflammatory response. Overproduction of proinflammatory cytokines can lead to an amplified and dysregulated secondary response. This hyper-inflammatory state, with the loss of normal immune homeostasis, ultimately causes tissue damage and organ dysfunction. A delicate balance exists between mounting a sufficient immune response to clear invading microbes and a negative feedback system to prevent inflammation-induced pathology. As a form of protection a period of immune hypo-responsiveness, also known as endotoxin tolerance, can occur with repeated LPS stimulation and is associated with the reduced survival seen in patients with septic shock [25].

Bacteria that cause diseases in humans can be divided into two broad categories, extracellular pathogens and intracellular pathogens [26,27]. Intracellular pathogens invade host cells and have the ability to survive and replicate within the cell [26]. In contrast, extracellular pathogens have developed ways to survive outside of host cells, as phagocytic uptake of these bacteria results in their rapid elimination [27]. These diverse strategies, used by bacteria to cause diseases, denote that the host has to develop a broad arsenal of defense mechanisms to protect themselves, for example the existence of both extracellular and intracellular PRRs as well as PRR-triggered immune responses [5,20,21,28,29]. To date, at least five major classes of PRRs have been identified, which include two families of membrane-bound PRRs: Toll-like receptors (TLRs) and C-type lectin receptors (CLR), and three families of cytoplasmic PRRs: the nucleotide binding and oligomerization domain (NOD)-like receptors (NLRs), retinoic acid-inducible gene (RIG)-I-like receptors (RLRs) and absence in melanoma 2 (Aim 2)-like receptors [28-30]. Among these PRRs, the TLRs and NLRs are two major classes of PRRs that are primarily responsible for the recognition of molecular structures of bacterial origin [30-33].

### TLR signaling and related intracellular signal transduction pathways in response to microbial infection

The transmembrane TLRs, with an extracellular domain involved in bacterial ligand recognition, are the most widely described PRRs. TLR1, TLR2, TLR4, TLR5, TLR6, and TLR10 are located at the extracellular surface, whereas TLR3, TLR7, TLR8, and TLR9 are located in the endoplasmic reticulum and endosomes [21,30,32,34]. TLRs were initially investigated in *Drosophila* [35], which has no adaptive immune system [36]. Eleven TLRs have been discovered in humans and thirteen in mice [37] and they, or their homologues, are found in all multicellular organisms [38]. Species differences do occur in the TLRs, which complicates attempts at cross-species direct comparisons. TLR2 and TLR4, which are expressed on the cell surface, are perhaps the most widely investigated of the TLR family and the only TLRs shown to be responsive to microbial ligands [39]. LPS or endotoxin, derived from gram-negative bacteria almost exclusively activates its primary receptor TLR4, one of the most studied pathways in host innate immunity against gram-negative bacterial infection. TLR4, initially named hToll was discovered in the 1990’s, when Hoshino and colleagues, using TLR4-deficient mice, demonstrated the hypo-responsiveness of these animals to LPS stimulation [40], thus confirming the pivotal role of TLR4 in the response to LPS. TLR4-deficient mice have been shown to be susceptible to gram-negative bacterial infection. In addition, specially bred mice that exclusively expressed TLR4 on endothelial cells were found to be more efficient at clearing *Escherichia coli* infection [41]. Smirnva et al., on examining DNA from patients with meningococcal disease found that a variant in the TLR4 gene is associated with an increased susceptibility to meningococcal septicemia [42]. Genetic variants in the TLR4 have also been linked to gram-negative bacterial infection in neonates [43]. TLR2, on the other hand, forms heterodimers TLR2/TLR1 and TLR2/TLR6 with either TLR1 or TLR6, and is a functional receptor for components of gram-positive bacteria including LTA, peptidoglycan (PGN) and bacterial lipopeptides, thus being responsible for the detection of gram-positive bacteria [44-46]. TLR2-deficient mice are highly susceptible to gram-negative *Staphylococcus aureus* infection, with significantly attenuated TNF-α and IL-6 production [47]. TLR5 and TLR9 recognize flagellin of bacteria flagella and bacterial CpG-DNA [21,48], respectively.

Upon engagement with their specific ligands, TLRs activate several intracellular signaling pathways. Signaling by TLRs in humans involves a family of five adaptor proteins, which interact with downstream protein kinases that ultimately lead to the activation of transcription factors including nuclear factor-κB (NF-κB) and members of the interferon (IFN)-regulatory factor (IRF) family. The Toll/interleukin-1 (IL-1) receptor (TIR) domain, which is unique to the TLR system, is the key signaling domain for not only TLRs, but also the adaptor protein. These five adaptor proteins include myeloid differentiation factor 88 (MyD88), MyD88 adaptor-like (MAL) protein, TIR-domain-containing adaptor protein inducing IFN-β (TRIF), TRIF-related adaptor molecule (TRAM) and sterile-α and armadillo-motif-containing protein (SARM) [49]. MAL, TRIF and TRAM are also known as TIR domain-containing adaptor protein (TIRAP), TIR-containing adaptor molecule-1 (TICAM1) and TICAM2. All TLRs (except TLR3) activate the MyD88 pathway, which results in the activation predominantly of the downstream NF-κB and mitogen-activated protein kinase (MAPK) signaling pathways, and ultimately leads to the production of inflammatory cytokines. Upon stimulation, MyD88 recruits IL-1 receptor-associated kinase (IRAK) family to TLRs and IRAK1 then associates with TNF receptor-associated factor 6 (TRAF6). This subsequently leads to the activation of NF-κB as well as MAPKs including p38, c-Jun NH2-terminal kinase (JNK) and extracellular signal-related kinase 1/2 (ERK1/2) [50]. Macrophages and DCs isolated from MyD88-deficient mice have been shown to be unable to respond to certain TLR ligands including TLR2, TLR5, TLR7 and TLR9 [51], indicating that these TLRs are fully dependent on the MyD88 signaling in order to activate the NF-κB signaling pathway. These cells however can remain somewhat responsive to LPS stimulation through a MyD88-independent pathway. TLR3 and TLR4 can activate a MyD88-independent/TRIF-dependent pathway, which allows for the activation of NF-κB and IRF3, and induction of IFN-β. TRIF (also known as TICAM1) activates TRAF3 and TRAF6, and signaling from TRAF3 induces IRF3 activation which allows for the production of IFN-β. Mice lacking TRIF fail to generate a type I IFN response to LPS stimulation, though their ability to activate the NF-κB and MAPK signaling pathways is preserved [52]. TRAM links TRIF to TLR4 and studies have shown TLR4 to possess the most complex signaling mechanism of all the TLRs, as TLR4 is the only member of the TLR family that recruits four adaptor proteins MyD88, MAL, TRIF and TRAM and activates two signaling pathways, namely the MyD88- and TRIF-dependent pathways [53].

### NOD signaling and related intracellular signal transduction pathways in response to microbial infection

NLRs are the newly discovered PRRs and are located in the intracellular cytoplasm [31,33,54-56]. Currently, 23 NLR proteins have been described in humans, and there are at least 34 TLR genes in mice [57]. NLR proteins are expressed by most human immune cells as well as a variety of non-immune cells including astrocytes, microglial cells, osteoblasts, myofibroblasts and epithelial cells [58]. This family of proteins is characterized by a tripartite structure consisting of i) a variable downstream protein-protein interaction domain, defined as the caspase activation and recruitment domain (CARD), a pyrin domain (PYD) or the baculovirus inhibitor repeat (BIR) domain; ii) a centrally located NOD domain or the central NACHT nucleotide-binding domain that facilitates oligomerization during activation and; iii) a C-terminal leucine-rich repeat (LRR) domain that detects PAMPs [33,54,55]. The LRR domain is pivotal in NLR activation following stimulation with bacterial products [59]. Oligomerisation of the central NACHT nucleotide-binding domain is another crucial step in NLR activation, with this step enabling the NF-κB mediated inflammatory response [60].

In contrast to the TLR family, the function of the majority of NLRs is poorly defined. However, NOD1 and NOD2, which play an important role in the intracellular recognition of invading pathogenic bacteria, are the two most comprehensively investigated members of the NLR family and have been shown to sense different structural core motifs derived from PGN, a cell wall component present in both gram-positive and gram-negative bacteria [61-64]. NOD1 is ubiquitously expressed in both stromal and hematopoietic cells [65], whereas NOD2 is only expressed in leukocytes including monocytes/macrophages and DCs, and in epithelial cells upon inflammatory stimulation [66]. NOD1 recognizes a PGN-related molecule containing the amino acid meso-diaminopimelic acid (meso-DAP) that is produced by most gram-negative and certain gram-positive bacteria [61,62], whereas NOD2 senses muramyl dipeptide (MDP) that is a conserved PGN motif present in all gram-positive and gram-negative bacteria [63,64]. Similar to TLR signaling, once activated, NOD1 and NOD2 enable their CARD domain to recruit and activate the adaptor protein, serine threonine kinase RICK, also known as receptor interacting protein 2 (RIP2). Activation of RIP2 is essential for the recruitment of TAK1, a kinase required for the activation of both the MAPK and IKK complex [67]. Subsequently, RIP2-induced activation of the IKK complex results in the phosphorylation and degradation of the inhibitor of κBα (IκBα), a critical step that allows for the nuclear translocation of NF-κB. The IKK complex also activates the MAPK family. Activation of the downstream NF-κB and MAPK signal transduction pathways stimulates the transcription and production of inflammatory cytokines and chemokines, which drive the host innate immune response to different infectious microbes. NOD1 not only activates NF-κB, but also MAPK JNK as seen in infection with *Shigella flexneri* [68]. *Streptococcus pneumonia*, another intracellular pathogen upregulates the expression of NOD1 and NOD2, with NOD2 playing an important role in the activation of downstream signaling molecules, which coordinate the activation of NF-κB [69].

Bacteria can activate NOD signaling by two different mechanisms; the T3SS, which has been identified in a number of gram-negative bacteria, is related to the flagellum and its main function is to deliver effector molecules across the cellular membrane of the host, whereas the T4SS, which has been identified in both gram-positive and gram-negative bacteria, is related to the bacterial conjugation system and can deliver DNA and proteins to the host cell [70]. *Helicobacter pylori* strains with functional T4SS, which delivers PGN intracellularly, were identified as potent activators of NOD1 [71]. NOD1-deficient mice are susceptible to infection with *Staphylococcus aureus* [72] and *Helicobacter pylori* [71]. Macrophages derived from NOD1-deficient mice are hyporesponsive to meso-DAP [61]. NOD2-deficient mice show enhanced susceptibility to *Toxoplasma gondii* infection [73]. Aberrant NOD2 signaling is strongly implicated in Crohn’s disease [74], whereby a defect in the LRR domain leads to an abnormal response to stimulation by bacterial components [63]. It is postulated that the normal hypo-responsiveness that exists to gut microflora becomes deranged in Crohn’s disease, causing an excessive inflammatory response to antigens within the microflora [75]. RIP2-deficient mice display an inability to respond to bacterial infection, underlining the importance of this molecule in NOD signaling [76]. RIP2-deficient mice also display impaired MAPK activation and attenuated cytokine responses. Stimulation of either NOD1 or NOD2 fails to activate the NF-κB pathway in fibroblasts derived from mice deficient in RIP2 and furthermore, RIP2-deficient mice display enhanced susceptibility to *Listeria monocytogenes* infection [77].

### Interaction and coordination between TLR and NOD signaling in innate immunity against microbial infection

The presence of PRRs located in multiple compartments of host cells raises questions about the reason for the development of microbial sensors with different cellular localizations. It is generally acknowledged that the location of the PRRs can dictate the type of bacteria they sense, for example, the cell surface PRRs such as the TLRs are activated primarily by extracellular bacteria, whereas the cytoplasmic PRRs like the NLRs, in particular NOD1 and NOD2, are responsible for sensing intracellular bacteria that are capable of breaching the plasma membrane and entering the cytoplasm [28,29]. However, emerging evidence has suggested that this is an inaccurate classification for extracellular and intracellular PRRs [69,72,78-80]. Indeed, many extracellular bacteria such as *Staphylococcus aureus* and *Streptococcus pneumonia* can be sensed by the cytoplasmic PRRs, NOD1 and NOD2 [69,72,78]. Conversely, TLR9, which senses CpG-containing DNA fragments, is responsible for the recognition of both extracellular and intracellular bacteria [79,80]. Furthermore, it has been reported that the maturation of bacteria-containing phagosomes is induced and accelerated in a TLR-dependent manner [81], suggesting that TLR signaling controls the maturation of phagosomes and destruction of captured microbial pathogens, including not only the extracellular bacteria but also the intracellular bacteria. It is more likely, therefore, that the role of extracellular and intracellular PPRs is to work together in order to induce an efficient innate immune response for the eradication of invading microbial pathogens, including both extracellular and intracellular bacteria from the body.

TLRs and NOD1/NOD2 collaborate with one another in mounting and balancing an efficient innate immune response to microbial pathogens. Both TLRs and NOD1/NOD2 respond to different PAMPs, which in some cases can be delivered by the same bacteria. Most TLRs activate the downstream NF-κB and MAPK pathways through recruitment and activation of their adaptor protein MyD88, whereas NOD1 and NOD2 activate the downstream NF-κB pathway through recruitment and activation of their adaptor proteins RIP2 and CARD9. Much investigation has been undertaken over the last number of years to elucidate the crosstalk that exists at different levels between the TLRs and NLRs. The TLR and NRL pathways can work synergistically in the host innate immune response to bacterial infection. Co-stimulation of human monocytes and DCs with the NOD1 agonist MtriDAP, the NOD2 agonists MDP and MtriLYS, and the TLR4 agonist LPS leads to enhanced production of inflammatory cytokines [82]. RIP2 is essential for activation of both NOD1- and NOD2-mediated signaling pathways; however, RIP2-knockout mice have defects in TLR2, TLR3 and TLR4 signaling [83]. RIP2-deficient mice have been shown to be more susceptible to pulmonary infection with *Chlamydophila pneumonia* [84] and are also more susceptible to infection with *Listeria monocytogenes* [85]. While NOD2 is essential for the recognition of MDP [86], MDP can increase the sensitivity of cells to LPS stimulation up to three-fold [87]. TLRs and NOD1/NOD2 also work synergistically in the production and maturation of IL-1β [88]. NOD2 is involved in the cleavage of pro-IL-1β to its active form by caspase-1, while TLRs, through the NF-κB pathway, allow for induction of pro-IL-1β [78].

Several mechanisms involved in the crosstalk between TLRs and NOD1/NOD2 have been proposed, which include upregulation of MyD88 expression in MDP-stimulated cells [89] and modulation of TLR signaling by the interaction of NOD2 and TAK1, a downstream molecule in the TLR signaling pathway [90]. The ability of MDP to augment the reaction of human monocytes and DCs to LPS stimulation is well documented [82]. LPS treatment stimulates NOD2 expression and TLR stimulation enhances the NOD2 response [91], through either upregulation of NOD2 or by induction of pro-IL-1β [92]. The essential roles of both TLR and NOD signaling pathways in the activation of IL-1β may act as a safeguard against excessive cytokine production. There appears to be a lack of cross-tolerisation between the TLR and NOD signaling pathways, with macrophages that are tolerant to the TLR4 agonist LPS remaining responsive to the NOD1 agonist mesoDAP and the NOD2 agonist MDP [93]. This may serve as an important backup mechanism for the host innate immune system in conditions where the tolerisation to TLR-associated signaling occurs. Cells also develop tolerance to NOD2 signaling with repeated exposure to the NOD2 agonist MDP [94]. This process is thought to be mediated by the rapid degradation of NOD2 protein, though these cells remain competent to stimulation with TLR ligands [94]. A separate experiment confirmed the ability of NOD2-deficient mice to react to TLR ligands despite having an impaired ability to mount a Th2-type response following stimulation with MDP [95]. This lack of cross-tolerisation may serve to preserve the host ability to mount an immune response to bacteria via NOD signaling, even in the TLR-tolerised state. It is important to note that some published papers did describe cross-tolerisation following stimulation of immune cells with NOD1/NOD2 agonists followed by TLR agonists; however, this effect may be due to impurities within the agonists used, the timing of pretreatment, or the type of cells used in the experiment.

### Novel therapeutic strategies for treatment of sepsis by targeting both TLR and NOD signaling

Over the past twenty years significant breakthroughs have been encountered in our understanding of the molecular mechanism(s) responsible for PRRs, PAMPS and the related signal transduction pathways involved in host innate immune responses to microbial infection. Targeted therapies can be directed at different phases of the host reaction to the invading microbial pathogens; however, there has been a dearth of potential therapies progressing to clinical trials and those that have, unfortunately so far proven ineffective. The immune system itself is a balance between proinflammatory and anti-inflammatory signals with multiple positive and negative feedback loops and complex signaling mechanisms. Both the hyper- and hypo-immune response must be taken into account with any potential therapies. Targeting individual molecules, however, is rife with problems.

#### Modulating TLR signaling

Interrupting different molecular mechanisms at play in TLR signaling has been made possible by the development of antagonistic molecules.

##### Targeting TLR4

High mobility group box protein-1 (HMGB1), released by innate immune cells, signals through TLR4. Reduced HMGB1 levels in caspase-1-deficient mice correlate with the resistance of these mice to LPS-associated lethality [96]. Anti-HMGB1 antibodies ameliorated the severity of sepsis in a cecal ligation and puncture (CLP)-induced polymicrobial sepsis model in rat [97]. Eritoran, an antagonist of the LPS-MD2-TLR4 complex, which blocked LPS-induced inflammatory cytokine response in vitro and in vivo, failed to produce any discernable benefit in reducing the mortality of patients with severe sepsis in a randomized, double-blind, and placebo-controlled phase 3 clinical trial [98]. Anti-TLR4 antibodies have been investigated in animal models of sepsis, with a reported benefit in certain studies [99,100], though a recent paper suggested no survival benefit in an *Escherichia coli*-induced abdominal sepsis model in mice [101]. TLR4 signaling is dependent on MyD88, which has been examined as an exciting potential therapeutic target for the treatment of sepsis. Research has shown that blocking the MyD88 adaptor protein substantially attenuates the IL-1β-driven inflammatory cytokine response to *staphylococcal* enterotoxin [102]. BAY 11–7082, an anti-inflammatory drug, which targets the MyD88 signaling pathway and potently inhibits NLRP3 activity [103], has not yet advanced to clinical trials. TAK-242 is a small molecule specific and selective inhibitor of TLR4. It binds to the TIR domain of TLR4 and also inhibits the binding of TIRAP and TRAM to TLR4, thus attenuating the production of LPS-induced inflammatory mediators [104]. In a murine model of *Escherichia coli*-induced peritonitis, a significant survival advantage was seen in mice treated with both TAK-242 and ceftazidime an hour following bacterial challenge [105]. Additionally, administration of TAK-242 strongly attenuated serum levels of proinflammatory cytokines in a murine endotoxic shock model and protected mice against LPS-induced lethality when mice were treated one hour before and 4 hours after LPS challenge [106]. Although TAK-242 showed great promise in experimental models of sepsis [107], it was withdrawn due to a lack of efficacy in a phase 3 clinical trial [108,109]. Despite the disappointing results seen in clinical trials with TLR4 antagonists, much research and excitement still persists in this field.

##### TLR2 antagonists

T2.5, an anti-TLR2 monoclonal antibody, inhibited inflammatory mediators and prevented lethal shock in a murine model of sepsis using *Bacillus subtilis* [110]. When T2.5 was used in combination with a TLR4-MD2 antagonist up to 4 hours after infection with *Escherichia coli* or *Salmonella enterica*, the survival benefit in mice was even greater [111].

##### TLR3 antagonists

In addition to the recognition of viral double-stranded RNA, TLR3 also senses DAMPs, which tend to be produced as a result of cellular damage or tissue injury in response to inflammation. A study in murine models of both cecal ligation-induced gut ischemia and CLP-induced septic peritonitis revealed that an anti-TLR3 neutralizing antibody ameliorated tissue injury associated with gut ischemia and also significantly improved survival in mice challenged with CLP-induced polymicrobial sepsis [112].

#### Modulating NOD signaling

In contrast to the TLR signaling, NOD signaling is less well-characterized as a therapeutic target during microbial infection and sepsis. Several studies have examined the role of NOD agonists in abrogating the severity of sepsis associated with bacterial infection. In a murine model of *Escherichia coli* bacteraemia and sepsis, there was disease abrogation in animals treated with MDP and tuftsin, two compounds acting in an immunomodulatory fashion by stimulating bacterial phagocytosis and inflammatory cytokine release, thereby enhancing host defense [113]. The NOD2 agonist MDP is too pyrogenic to be used in the clinical setting and therefore several MDP derivatives without this side-effect have been investigated. The MDP derivative, murabutide, has been examined for its role in enhancing the host innate immune response to microbial infection, although a trial did not find significantly higher levels of circulating inflammatory cytokines in healthy volunteers [114]. Nevertheless, NOD agonists, when used prophylactically as adjuncts to antibiotics, substantially improve survival in animal models of microbial sepsis [115]. A patent currently exists on the use of a TLR9 agonist/TLR4 antagonist and/or NOD2 agonist for the treatment of systemic sepsis and necrotizing enterocolitis [116]. Activation of TLR9 inhibits TLR4 signaling in enterocytes, leading to abrogation of the inflammatory reaction, whereas activation of NOD2 also led to a reduction in TLR4 signaling in treated enterocytes. TLR9 agonists promote a type I IFN response [117], thus enhancing bacterial phagocytosis and killing in experiments using *Salmonella typhimurium* [118] and *Streptococcus pneumonia* [119].

## Conclusion

Sepsis is a leading cause of death in intensive care units worldwide. Factors associated with increased risk of sepsis include male race, extremes of age, comorbid medical conditions and people of African-American descent. Our knowledge of the molecular mechanisms involved in sepsis continues to grow. The innate immune system is vital in initiating the host defense against microbial pathogens; however, a dysregulated inflammatory response triggered by the innate immunity can be deleterious to the host. To date, clinical trials targeting TLRs and the related signaling pathways have failed. Several reasons are postulated for the lack of success including but not limited to the following: the extraordinary heterogeneity that exists in the septic population, comorbid conditions affecting individual patients, underpowered studies and the range of diagnostic criteria used in sepsis (Table 1). Also of importance is the fact that most animal models of human sepsis are deeply flawed. The systemic inflammatory response in mice is very different to that seen in humans, and is compressed into a few days whereas the human course can often follow a more protracted course, possibly related to the significant supportive care adjuncts that are in use in intensive care units [120]. These factors contribute to difficulties in translating promising preclinical targets in animal experiments into practical applications in human sepsis. In this review we have attempted to describe the roles of TLR and NOD signaling in host defense against microbial infection and the potential therapeutic strategies targeting both TLR and NOD signaling. Our understanding of the interplay between TLR and NOD signaling is rapidly expanding, contributing to the potential for future targeted therapies aimed at ameliorating the global morbidity and mortality related to microbial sepsis. At present, no clinical trials are actively examining TLR or NOD targeted therapies. These authors remain hopeful that successful targeted therapies will be developed in the future.

## Abbreviations

ACCP: American College of Chest Physicians
Aim 2: Absence in melanoma 2
ATS: American Thoracic Society
BIR: Baculovirus inhibitor repeat
CARD: Caspase activation and recruitment domain
CLP: Cecum ligation and puncture
CLR: C-type lectin receptors
DAMPs: Damage-associated molecular patterns
DCs: Dendritic cells
EPIC: Extended Study on the Prevalence of Infection in Intensive Care
ERK1/2: Extracellular signal-related kinase 1/2
ESICM: European Society of Intensive Care Medicine
IFN: Interferon
IκBα: Inhibitor of κBα
IL-1: Interleukin-1
IRAK: IL-1 receptor-associated kinase
IRF: IFN-regulatory factor
JNK: c-Jun NH_2_-terminal kinase
LPS: Lipopolysaccharide
LRR: Leucine-rich repeat
LTA: Lipoteichoic acid
MAL: MyD88 adaptor-like
MAPK: Mitogen-activated protein kinase
MDP: Muramyl dipeptide
MyD88: Myeloid differentiation factor 88
NF-κB: Nuclear factor-κB
NLRs: NOD-like receptors
PAMPs: Pathogen-associated molecular patterns
PMNs: Polymorphonuclear neutrophils
PRRs: Pattern recognition receptors
PYD: Pyrin domain
RIG: Retinoic acid-inducible gene
RIP2: Receptor interacting protein 2
RLRs: RIG-I-like receptors
ROS: Reactive oxygen species
SARM: Sterile-α and armadillo-motif-containing
SCCM: Society for Critical Care Medicine
SIS: Surgical Infection Society
TICAM1: TIR-containing adaptor molecule-1
TIR: Toll/IL-1 receptor
TIRAP: TIR domain-containing adaptor protein
TLR: Toll-like receptor
TRAF6: TNF receptor-associated factor 6
TRAM: TRIF-related adaptor molecule
TRIF: TIR-domain-containing adaptor protein inducing IFN-β
